# Penetration of new antidiabetic medications in East Asian countries and the United States: A cross-national comparative study

**DOI:** 10.1371/journal.pone.0208796

**Published:** 2018-12-12

**Authors:** Kiyoshi Kubota, Yukari Kamijima, Yea-Huei Kao Yang, Shinya Kimura, Edward Chia-Cheng Lai, Kenneth K. C. Man, Patrick Ryan, Martijn Schuemie, Paul Stang, Chien-Chou Su, Ian C. K. Wong, Yinghong Zhang, Soko Setoguchi

**Affiliations:** 1 NPO Drug Safety Research Unit Japan, Tokyo, Japan; 2 Institute of Clinical Pharmacy and Pharmaceutical Sciences, College of Medicine, National Cheng Kung University, Tainan, Taiwan; 3 Japan Medical Data Center, Tokyo, Japan; 4 Duke Clinical Research Institute, Duke University School of Medicine, Durham, North Carolina, United States of America; 5 Centre for Safe Medication Practice and Research, Department of Pharmacology and Pharmacy, University of Hong Kong, Hong Kong, China; 6 Research Department of Practice and Policy, UCL School of Pharmacy, London, United Kingdom; 7 Janssen Research & Development, LLC, Titusville, New Jersey, United States of America; 8 Department of Epidemiology, Rutgers School of Public Health, New Brunswick, United States of America; Weill Cornell Medical College Qatar, QATAR

## Abstract

**Background:**

The number of patients with diabetes is increasing particularly in Asia-Pacific region. Many of them are treated with antidiabetics. As the basis of the studies on the benefit and harm of antidiabetic drugs in the region, the information on patterns of market penetration of new classes of antidiabetic medications is important in providing context for subsequent research and analyzing and interpreting results.

**Methods:**

We compared penetration patterns of dipeptidyl peptidase-4 (DPP-4) inhibitors in Taiwan, Hong Kong, Japan, and the United States. We used the Taiwan National Health Insurance Research Database, a random sample of the Hong Kong Clinical Data Analysis and Reporting System, the Japan Medical Data Center database, and a 5% random sample of the US Medicare database converted to the Observational Medical Outcomes Partnership’s Common Data Model to identify new users of oral antidiabetic medications. We standardized prevalence and incidence rates of medication use by age and sex to those in the 2010 Taiwanese population. We compared age, sex, comorbid conditions, and concurrent medications between new users of DPP-4 inhibitors and biguanides.

**Results:**

Use of DPP-4 inhibitors 1 year after market entry was highest in Japan and lowest in Hong Kong. New users had more heart failure, hyperlipidemia, and renal failure than biguanide users in Taiwan, Hong Kong, and the United States while the proportions were similar in Japan. In a country with low penetration of DPP-4 inhibitors (eg, Hong Kong), users had diabetes with multiple comorbid conditions compared with biguanidine users. In a country with high penetration (eg, Japan), the proportion of users with comorbid conditions was similar to that of biguanide users.

**Conclusions:**

We observed a marked difference of the penetration patterns of newly marketed antidiabetics in different countries in Asia. Those results will provide the basic information useful in the future studies.

## Introduction

According to the International Diabetes Federation, the number of people with diabetes worldwide will increase from 425 million in 2017 to 629 million in 2045. More than half are in the Asia-Pacific region [1]. Glucose control is an essential strategy in diabetes management aimed at the prevention of microvascular complications with modest benefit in lowering the risk of macrovascular disorders [2]. Antidiabetic medication is used for glucose control when lifestyle intervention alone is insufficient to control blood glucose at a target level. In the past 2 decades, new classes of antidiabetic medications have been developed and marketed, including meglitinides [3], thiazolidinediones [4], dipeptidyl peptidase-4 (DPP-4) inhibitors [5], incretin mimetics [6], and sodium-glucose cotransporter 2 inhibitors [7].

The benefit and harm profiles associated with these new classes of medications may differ between the West and Asia. For example, the risks of macrovascular disorders and all-cause mortality differ substantially between patients with diabetes in the West and those in Asia [8]. These differences increase the importance of further study of the safety and effectiveness of existing and new antidiabetic medications in Asia. To facilitate such studies, information on patterns of market penetration of new classes of antidiabetic medications and the characteristics of patients who are prescribed new medication classes compared to old ones across countries is important in providing context for subsequent research and analyzing and interpreting results.

The Asian Pharmacoepidemiology Network (AsPEN) is a special interest group of the International Society of Pharmacoepidemiology for developing and advancing multinational database research in pharmacoepidemiology in the Asia-Pacific region [9]. The Surveillance of Health Care in Asian Network (SCAN) project is an activity of AsPEN intended to provide better understanding of the health and health care resource use of the populations covered in each participating site database in AsPEN [10]. In the current study, we investigated the penetration of DPP-4 inhibitor use in Taiwan (marketed in March 2009), Hong Kong (November 2007), and Japan (December 2009). For reference, we also examined prescribing patterns for the same medications in the United States (marketed in October 2006).

## Materials and methods

### Study design

We conducted a descriptive study where the annual prevalence and incidence of the use of antidiabetics were compared between Taiwan, Hong Kong, Japan and the US with a particular attention to the use of DPP-4 inhibitors and biguanides.

### Databases

We used data for 1 million patients collected between 2001 and 2010 from the Taiwan National Health Insurance Research Database (NHIRD) [11]; a random sample of data collected between 2008 and 2013 from the Hong Kong Clinical Data Analysis and Reporting System (CDARS) [12]; data collected between 2006 and 2014 from the Japan Medical Data Center (JMDC) [13]; and data collected between 2006 and 2011 from a 5% random sample of Medicare beneficiaries in the United States [14]. The NHIRD, the JMDC, and the Medicare 5% sample are claims databases, whereas the CDARS is a computerized clinical management system (ie, electronic health record). The NHIRD is a representative sample of the entire population of Taiwan, in contrast to the CDARS database, which includes inpatient and outpatient data for all public hospitals managed by the Hong Kong Hospital Authority. The JMDC database consists of corporate health insurance claims and includes data for persons of working age and their family members in the same household. Because the JMDC database reflects a working population, persons 65 years or older are not well represented. For example, the ratio of workers to their family members is 1.08 among those younger than 65 years, whereas the ratio is 1.02 among those 65 years or older, indicating that approximately half of older beneficiaries are still working and are likely to be healthier than older persons in the overall Japanese population. The US Medicare database consists mainly of data for persons 65 years or older, but also includes data for persons younger than 65 years who receive Social Security Disability Insurance [15] or have been diagnosed with end-stage renal disease.

The 4 databases were converted to the Observational Medical Outcomes Partnership’s Common Data Model (CDM) [16]. The research coordinating center in the Duke Clinical Research Institute (Durham, North Carolina, USA) developed analytic SAS program code based on the Common Data Model for the SCAN project and distributed the code to each study site. Each site then ran the SAS routine and returned summary results (without individual-level information) to the coordinating center.

### Participants

We identified patients with at least 1 dispensing of an oral antidiabetic medication, including biguanides, sulfonylureas, alpha-glucosidase inhibitors, meglitinides, thiazolidinediones, and DPP-4 inhibitors and all patients with type 2 diabetes and compared the prevalence and incidence rates of the use of the major classes of antidiabetic medications. We defined new (incident) use for each medication class as at least 1 dispensing of antidiabetic medication of a particular class after 6 months or longer of no dispensing of any medication in the class. We further classified new use of a medication class as “single” (ie, new use without concurrent use of another antidiabetic medication), “dual” (ie, new use with concurrent use of 1 other class of antidiabetic medication, and “multiple” (ie, new use with concurrent use of 2 or more other classes of antidiabetic medication). For new users, we identified concurrent medications and comorbid conditions in the 6 months preceding initiation of the antidiabetic medication of interest. We standardized the prevalence and incidence rates of medication use to the age and sex of the Taiwan population in 2010. To characterize new users of DPP-4 inhibitors, we compared the patients with new users of biguanides, because biguanides are considered a first-line medication in most countries.

### Analysis

We compared the distributions of age, sex, and other characteristics between new users of DPP-4 inhibitors and new users of biguanides and estimated the standardized difference [17]. Although the number of patients with type 2 diabetes in the current study differed between countries, standardized differences are not sensitive to sample size. Standardized differences of greater than 0.1 (or smaller than –0.1) are typically considered meaningful [18].

We analyzed the data in 2 age groups—patients younger than 65 years and patients 65 years or older—because the former group in the Medicare database and the latter group in the JMDC database are not representative of the general population. However, we combined the 2 age groups where appropriate.

### Relevant guidelines

We conducted the study according to the Guidelines for Good Pharmacoepidemiology Practices (GPP) [19] and reported the study results according to the STROBE guidelines [20].

This study was approved by the institutional review board of the Duke University Health System and by the Institutional Review Board of National Cheng Kung University Hospital in Taiwan, the Institutional Review Board of the University of Hong Kong/Hospital Authority Hong Kong West Cluster in Hong Kong, and the ethics committee of the Tokyo University Graduate School and Faculty of Medicine in Japan. All of the data of Medicare 5% sample, NHIRD, CDARS, and JMDC were fully anonymized before we accessed them. No national regulation and laws in Taiwan, Hong Kong and Japan apply to sending non-individual-level information abroad.

## Results

Table 1 shows the age and sex distributions of patients with type 2 diabetes. The proportions of patients 65 years or older were 40.5% in Taiwan, 54.9% in Hong Kong, 19.2% in Japan, and 77.2% in the United States.

**Table 1:** Age and sex distribution of patients with type 2 diabetes in Taiwan, Hong Kong, Japan, and the United States.

Characteristic	Taiwan NHIRD, 2001–2010 (n = 88,042)	Hong Kong CDARS, 2008–2013 (n = 7493)	Japan JMDC, 2006–2014 (n = 69,895)	US Medicare 5%, 2006–2011 (n = 343,166)
Men, No. (%)	43,711 (49.6)	3630 (48.4)	47,689 (68.2)	134,005 (39.0)
Age group, No. (%)				
0–18 y	972 (1.1)	11 (0.1)	797 (1.1)	0
19–34 y	4473 (5.1)	103 (1.4)	3045 (4.4)	3901 (1.1)
35–44 y	8548 (9.7)	319 (4.3)	8900 (12.7)	10,386 (3.0)
45–54 y	18,831 (21.4)	1191 (15.9)	17,449 (25.0)	24,955 (7.3)
55–64 y	19,568 (22.2)	1754 (23.4)	26,315 (37.6)	39,125 (11.4)
65–69 y	18,220 (20.7)	1379 (18.4)	9482 (13.6)	86,623 (25.2)
70–79 y	13,339 (15.2)	1780 (23.8)	3811 (5.5)	105,307 (30.7)
80–89 y	3757 (4.3)	832 (11.1)	89 (0.1)	60,791 (17.7)
> 90 y	334 (0.4)	124 (1.7)	7 (0.01)	12,078 (3.5)

Abbreviations: CDARS, Clinical Data Analysis and Reporting System; JMDC, Japan Medical Data Center; NHIRD, National Health Insurance Research Database.

**Fig 1:**
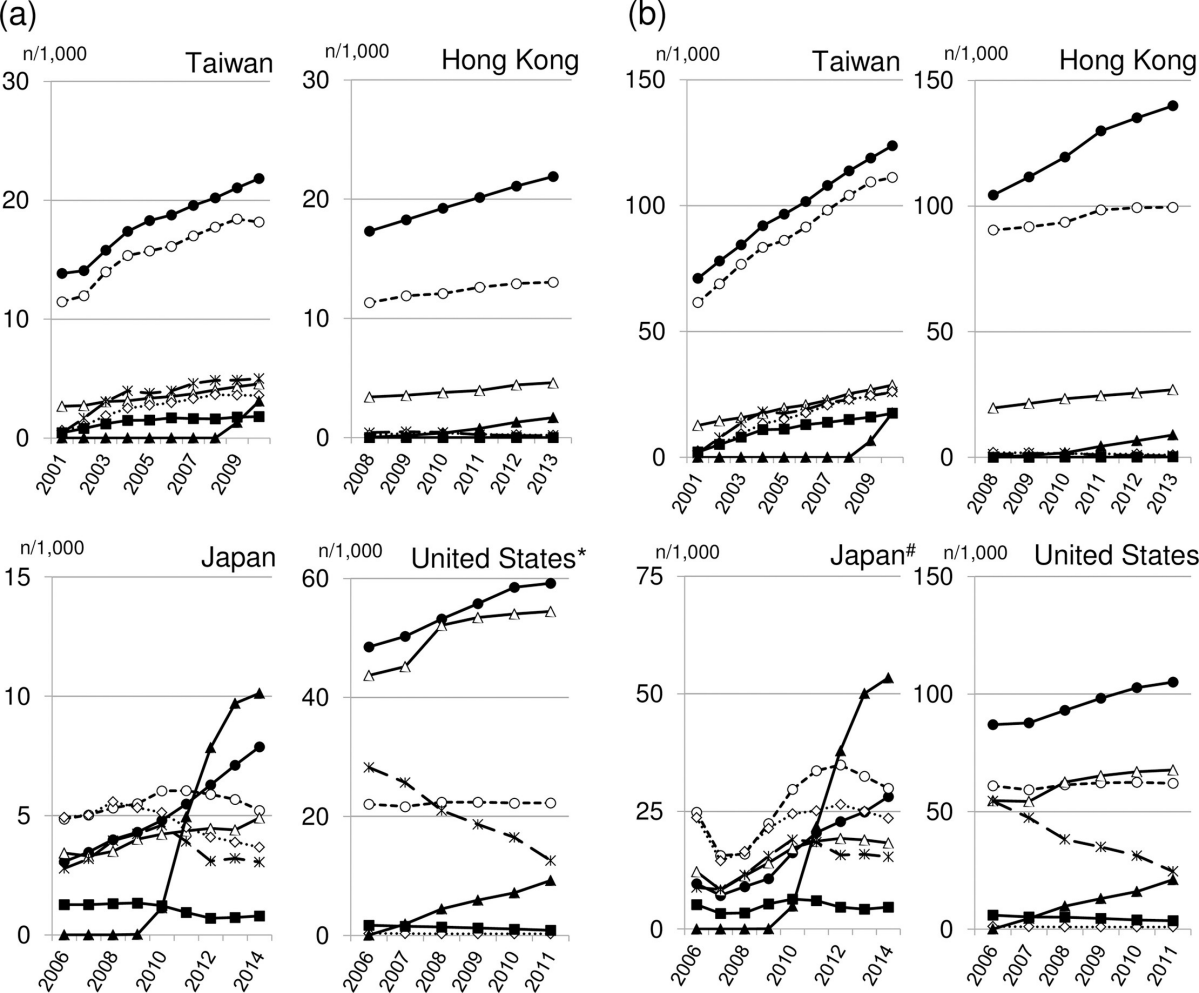
Prevalence per 1000 patients of the use of biguanides (●), sulphonyl urea (○), insulin (△), thiazolidinediones (✕), alpha-glucosidase inhibitors (◇), meglitinides (■) and dipeptidyl peptidase 4 (DPP-4) inhibitors (▲) in Taiwan, Hong Kong, Japan, and the United States. Panel A shows patients younger than 65 years; Panel B shows patients 65 years or older. * The study population is not representative of the corresponding age group in the general population in the United States (see text for details). † The study population is not representative of the corresponding age group in the general population in Japan (see text for details).

Fig 1 shows the prevalence of use of 7 classes of antidiabetic medications (ie, biguanides, sulfonylureas, insulin, thiazolidinediones, alpha-glucosidase inhibitors, meglitinides, and DPP-4 inhibitors). The age- and sex-adjusted prevalence of DPP-4 inhibitor vs. biguanidine per 1000 patients about 1 year after marketing of DPP-4 inhibitors was 3.1 vs 21.8 in Taiwan (in 2010), 0.2 vs 18.3 in Hong Kong (in 2009), 4.9 vs 5.5 in Japan (in 2011), and 4.4 vs 53.2 in the United States (in 2008) among patients younger than 65 years (Fig 1A) and 17.5 vs 123.7 in Taiwan, 0.8 vs 111.6 in Hong Kong, 21.8 vs 20.5 in Japan, and 9.8 vs 93.1 in the United States among patients 65 years or older (Fig 1B).

**Fig 2:**
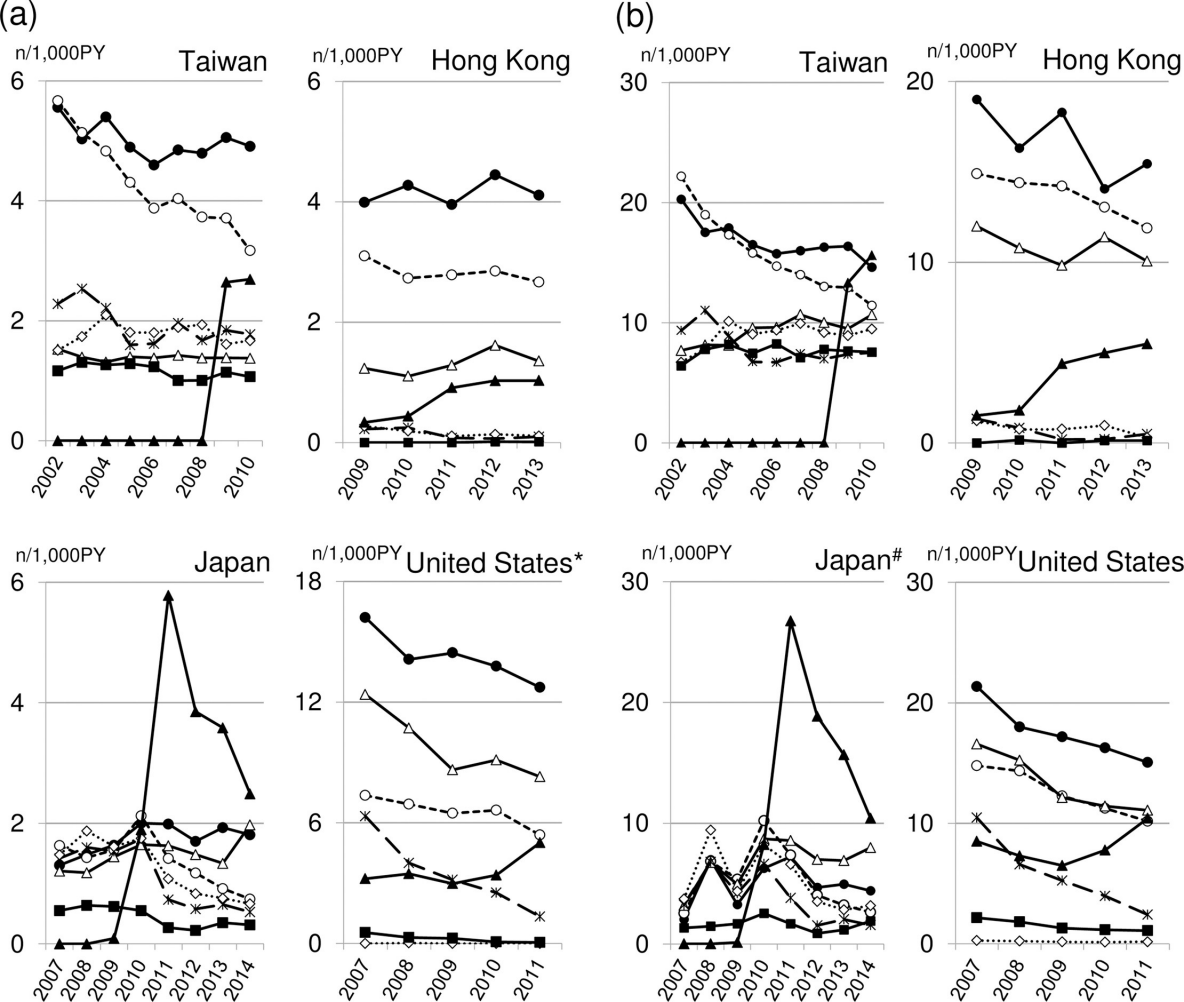
Incidence per 1000 patient-years of the use of biguanides (●), sulphonyl urea (○), insulin (△), thiazolidinediones (✕), alpha-glucosidase inhibitors (◇), meglitinides (■) and dipeptidyl peptidase 4 (DPP-4) inhibitors (▲) in Taiwan, Hong Kong, Japan, and the United States. Panel A shows patients younger than 65 years; Panel B shows patients 65 years or older. † The study population is not representative of the corresponding age group in the general population in the United States (see text for details). # The study population is not representative of the corresponding age group in the general population in Japan (see text for details).

Fig 2 shows the age- and sex-adjusted incidence rates of new use of each of the 7 classes of antidiabetic medications. The incidence rate of DPP-4 inhibitors vs biguanides per 1000 patient-years was 2.7 vs 4.9 in Taiwan, 0.3 vs 4.0 in Hong Kong, 5.8 vs 2.0 in Japan, and 3.5 vs 14.1 in the United States among patients younger than 65 years (Fig 2A) and 15.6 vs 14.6 in Taiwan, 1.5 vs 19.0 in Hong Kong, 26.8 vs 7.2 in Japan, and 7.3 vs 18.0 in the United States among patients 65 or older (Fig 2B).

Table 2 shows the distributions of age, sex, and other characteristics of new users of DPP-4 inhibitors and biguanides across patients of all ages (The distributions of the 2 age groups are shown separately in S1 and S2 Tables). New users of DPP-4 inhibitors were in general older than new users of biguanides; a biguanidine was used as a single medication in more than 50% of new users of biguanides in Taiwan, Hong Kong, and the United States, but for approximately 30% in Japan. A DPP-4 inhibitor was used as a single medication in approximately 5% of new users of DPP-4 inhibitors in Taiwan and Hong Kong, but for 23% in United States and 37% in Japan. The proportion of patients with heart failure, hyperlipidemia, and renal failure among new users of DPP-4 inhibitors was higher than among new users of biguanides in Taiwan, Hong Kong, and the United States (standardized difference> 0.1), but the difference was not meaningful (standardized difference ≤0.1) in Japan. Similarly, the proportion of patients with concurrent use of beta-blockers, diuretics, and non-statin lipid-lowering drugs was much higher among new users of DPP-4 inhibitors than among new users of biguanides in Taiwan, Hong Kong, and United States (standardized difference> 0.1), but the difference was not meaningful (standardized difference ≤0.1) in Japan.

**Table 2:** Characteristics of new users of dipeptidyl peptidase-4 inhibitors and biguanides.

Characteristic	Taiwan			Hong Kong			Japan			United States		
	DPP-4 Inhibitor (n = 7497)	Biguanide (n = 48,447)	Std Diff	DPP-4 Inhibitor (n = 530)	Biguanide (n = 2667)	Std Diff	DPP-4 Inhibitor (n = 23,597)	Biguanide (n = 14,004)	Std Diff	DPP-4 Inhibitor (n = 33,420)	Biguanide (n = 76,132)	Std Diff
Men, No. (%)	3710 (49.5)	24,615 (50.8)	–0.03	249 (47.0)	1361 (51.0)		16,987 (72.0)	9902 (70.7)		12,622 (37.8)	30,220 (39.7)	
Age, mean (SD), y	61.7 (12.7)	56.6 (14.6)	0.35	63.8 (12.6)	62.2 (13.1)	0.12	54.3 (9.7)	51.8 (10.5)	0.24	70.9 (11.8)	69.1 (11.9)	0.15
Pattern, No. (%)^a^												
Single use	431 (5.7)	25,501 (52.6)	–0.94	17 (3.2)	1676 (62.8)	–1.19	8663 (36.7)	4118 (29.4)	0.15	7662 (22.9)	48,421 (63.6)	–0.81
Dual use	2508 (33.5)	20,242 (41.8)	–0.17	91 (17.2)	906 (34.0)	–0.36	7594 (32.2)	5267 (37.6)	–0.11	14,389 (43.1)	22,333 (29.3)	0.29
Multiple use	4558 (60.8)	2704 (5.6)	1.64	424 (80.0)	100 (3.7)	2.06	7340 (31.1)	4619 (33.0)	–0.04	11,369 (34.0)	5378 (7.1)	0.75
Concurrent antidiabetic medication, No. (%)												
Alpha-glucosidase inhibitor	1285 (17.1)	1616 (3.3)	0.62	34 (6.4)	15 (0.6)	0.47	4354 (18.5)	2691 (19.2)	–0.02	296 (0.9)	169 (0.2)	0.11
Biguanidine	4500 (60.0)	—	—	455 (85.8)	—	—	6949 (29.4)	—	—	14,913 (44.6)	—	—
DPP-4 inhibitor	—	220 (0.5)	—	—	33 (1.2)	—	—	4774 (34.1)	—	—	6240 (8.2)	—
Insulin	914 (12.2)	1520 (3.1)	0.45	109 (20.6)	172 (6.4)	0.50	2360 (10.0)	1700 (12.1)	–0.07	5705 (17.1)	8689 (11.4)	0.17
Meglitinide	670 (8.9)	1705 (3.5)	0.27	1 (0.2)	3 (0.1)	0.03	857 (3.6)	591 (4.2)	–0.03	1137 (3.4)	755 (1.0)	0.18
Sulfonylurea	4519 (60.3)	19,423 (40.1)	0.41	406 (76.6)	877 (32.9)	0.89	7020 (29.7)	4235 (30.2)	–0.01	10,678 (32.0)	14,423 (18.9)	0.31
Thiazolidinedione	1319 (17.6)	1440 (3.0)	0.67	29 (5.5)	12 (0.4)	0.45	3646 (15.5)	2003 (14.3)	0.03	6,897 (20.6)	7107 (9.3)	0.34
Comorbid conditions, No. (%)												
Asthma	364 (4.9)	2516 (5.2)	–0.01	1 (0.2)	13 (0.5)	–0.05	2157 (9.1)	1248 (8.9)	0.01	3614 (0.01)	8673 (11.4)	–0.02
Atrial fibrillation	162 (2.2)	641 (1.3)	0.08	10 (1.9)	24 (0.9)	0.10	491 (2.1)	197 (1.4)	0.05	4377 (13.1)	7901 (10.4)	0.09
COPD	230 (3.1)	1831 (3.8)	–0.04	1 (0.2)	20 (0.7)	–0.06	2364 (10.0)	1266 (9.0)	0.08	2261 (6.8)	5308 (7.0)	–0.01
Dementia	178 (2.4)	710 (1.5)	0.07	0	12 (0.4)	–0.07	74 (0.3)	21 (0.1)	0.04	1640 (< 0.1)	3526 (4.6)	0.01
Depression	336 (4.5)	1860 (3.8)	0.04	2 (0.4)	18 (0.7)	–0.04	1204 (5.1)	692 (4.9)	0.01	2323 (7.0)	5794 (7.6)	–0.02
Epilepsy	41 (0.5)	291 (0.6)	–0.01	0	2 (0.1)	–0.04	366 (1.6)	212 (1.5)	0.01	553 (1.7)	1518 (2.0)	–0.02
Heart failure	444 (5.9)	1695 (3.5)	0.13	19 (3.6)	26 (1.0)	0.22	2483 (10.5)	1252 (8.9)	0.05	7167 (< 0.1)	11,014 (14.5)	0.19
Hyperlipidemia	4098 (54.6)	17,484 (36.1)	0.38	29 (5.5)	67 (2.5)	0.18	14,883 (63.1)	8272 (59.1)	0.08	28,203 (84.4)	56,871 (74.7)	0.23
Hypertension	4368 (58.3)	20,877 (43.1)	0.31	58 (10.9)	188 (7.0)	0.15	12,390 (52.5)	6484 (46.3)	0.12	30,112 (90.1)	63,172 (83.0)	0.20
Malignant neoplasm	436 (5.8)	1998 (4.1)	0.08	7 (1.3)	36 (1.3)	0.00	4945 (21.0)	2680 (19.1)	0.05	4350 (13.0)	8523 (11.2)	0.06
Mood disorder	359 (4.8)	2047 (4.2)	0.03	2 (0.4)	18 (0.7)	–0.04	1281 (5.4)	740 (5.3)	0.00	2817 (8.4)	7423 (9.8)	–0.05
Myocardial infarction	66 (2.1)	578 (1.2)	0.08	4 (0.8)	14 (0.5)	0.04	1040 (4.4)	536 (3.8)	0.03	813 (2.4)	1409 (1.9)	0.04
Parkinson disease	356 (0.9)	325 (0.7)	0.02	0	5 (0.2)	–0.05	109 (0.5)	48 (0.3)	0.03	470 (1.4)	938 (1.2)	0.02
Pneumonia	54 (4.7)	1867 (3.9)	0.04	9 (1.7)	37 (1.4)	0.03	977 (4.1)	521 (3.7)	0.02	670 (2.0)	1413 (1.9)	0.01
Renal failure	591 (7.9)	840 (1.7)	0.39	13 (2.5)	11 (0.4)	0.24	352 (1.5)	120 (0.9)	0.05	3228 (9.7)	3138 (4.1)	0.24
Rheumatoid arthritis	82 (1.1)	424 (0.9)	0.02	0	0	—	713 (3.0)	342 (2.4)	0.04	1199 (3.6)	2581 (3.4)	0.01
Schizophrenia	54 (0.7)	439 (0.9)	–0.02	0	6 (0.2)	–0.05	298 (1.3)	187 (1.3)	0.00	651 (1.9)	2264 (3.0)	–0.07
Concurrent medications, No. (%)												
Anti-arrhythmic	222 (3.0)	1071 (2.2)	0.05	7 (1.3)	9 (0.3)	0.14	3236 (13.7)	1642 (11.7	0.06	2103 (6.3)	3039 (4.0)	0.11
Antidementia	191 (2.5)	861 (1.8)	0.05	4 (0.8)	12 (0.4)	0.06	33 (0.1)	7 (< 0.1)	0.03	1790 (5.4)	3285 (4.3)	0.05
Antidepressant	776 (10.4)	3667 (7.6)	0.10	23 (4.3)	107 (4.0)	0.02	811 (3.4)	517 (3.7	–0.02	9762 (29.2)	22,169 (29.1)	0.00
Anti-Parkinson	254 (3.4)	1770 (3.7)	–0.02	1 (0.2)	44 (1.6)	–0.12	154 (0.7)	75 (0.5)	0.03	1546 (4.6)	3476 (4.6)	0.00
Antipsychotic	553 (7.4)	3,47 (7.5)	0.00	32 (6.0)	178 (6.7)	–0.03	682 (2.9)	400 (2.9)	0.00	3010 (9.0)	8174 (10.7)	–0.06
Benzodiazepine	1301 (17.4)	7208 (14.9)	0.07	24 (4.5)	107 (4.0)	0.03	2583 (10.9)	1276 (9.1)	0.06	3038 (9.1)	5681 (7.5)	0.06
β-Blocker	2504 (33.4)	11,854 (24.5)	0.20	192 (36.2)	613 (23.0)	0.30	2127 (9.0)	982 (7.0)	0.07	15,455 (46.2)	28,544 (37.5)	0.18
Calcium channel blocker	3278 (43.7)	14,474 (29.9)	0.30	214 (40.4)	858 (32.2)	0.17	6081 (25.8)	2831 (20.2)	0.13	10,195 (30.5)	18,425 (24.2)	0.14
COPD medication	2511 (33.5)	16,950 (35.0)	–0.03	24 (4.5)	123 (4.6)	0.00	5379 (22.8)	2936 (21.0)	0.04	9308 (27.9)	19,914 (26.2)	0.04
Diuretic	2537 (33.8)	8941 (18.5)	0.38	85 (16.0)	192 (7.2)	0.31	1370 (5.8)	594 (4.2)	0.07	18,300 (54.8)	35,105 (46.1)	0.17
Non-statin lipid-lowering drug	927 (12.4)	2766 (5.7)	0.27	29 (5.5)	46 (1.7)	0.25	1457 (6.2)	735 (5.2)	0.04	3306 (9.9)	4966 (6.5)	0.13
NSAID	4308 (57.5)	28,906 (59.7)	–0.04	34 (6.4)	266 (10.0)	–0.12	6840 (29.0)	3721 (26.6)	0.05	7069 (21.2)	15,362 (20.2)	0.02
RAS inhibitor	1117 (14.9)	5961 (12.3)	0.08	245 (46.2)	475 (17.8)	0.68	961 (4.1)	479 (3.4)	0.04	15,111 (45.2)	27,833 (36.6)	0.18
Statin	3215 (42.9)	6105 (12.6)	0.81	235 (44.3)	533 (20.0)	0.57	7581 (32.1)	3715 (26.5)	0.12	21,181 (63.4)	36,388 (47.8)	0.31
Vitamin K antagonist	111 (1.5)	310 (0.6)	0.10	11 (2.1)	31 (1.2)	0.08	377 (1.6)	140 (1.0)	0.05	3029 (9.1)	5696 (7.5)	0.06

Abbreviations: Std Diff: standardized difference; COPD, chronic obstructive pulmonary disease; DPP-4, dipeptidyl peptidase-4; NSAID, nonsteroidal anti-inflammatory drug; RAS, renin-angiotensin system.

^a^ Single use refers to new use of a DPP-4 inhibitor or biguanidine without concurrent use or initiation of another antidiabetic medication. Dual use refers to new use of a DPP-4 inhibitor or biguanidine with concurrent use or initiation of 1 other antidiabetic medication. Multiple use refers to new use of a DPP-4 inhibitor or biguanidine with concurrent use or initiation of 2 or more other antidiabetic medications.

## Discussion

To our knowledge, ours is the first study to compare the penetration pattern of a new class of antidiabetic medications between multiple countries in Asia. Previous studies have examined patterns of medication use over several years in a single country^20^or in 2 countries [21].

In our study of medication prescribing patterns in Taiwan, Hong Kong, Japan, and the United States approximately 1 year after marketing of DPP-4 inhibitors began in each country, prevalence and incidence rates of DPP-4 inhibitor use were highest in Japan and lowest in Hong Kong. The proportions of comorbid heart failure, hyperlipidemia, and renal failure and the concurrent use of medications to treat those comorbid conditions among new users of DPP-4 inhibitors were higher than among new users of biguanides in Taiwan, Hong Kong, and the United States, but the differences were not meaningful in Japan. This finding suggests that DPP-4 inhibitors may be preferentially prescribed to more medically complex patients with diabetes except in Japan.

Different penetration patterns of a new class of antidiabetic medications may be, to some extent, explained by treatment guidelines and institutional and economic constraints on the use of new medications. In guidelines in the United States [22] and the European Union [2], metformin is indicated as the preferred initial pharmacological medication for type 2 diabetes. Guidelines published by the Japan Diabetes Society [23] indicate, “If good control cannot be achieved with one type of oral hypoglycemic agent, combination therapy with another drug having a different mode of action should be carried out”; however, no specific guidance is provided as to the medication preferred first. This may explain in part the high proportion of “single” use among new users of DPP-4 inhibitors and the observation that the proportion of comorbid conditions and concomitant medications was similar between new users of DPP-4 inhibitors and biguanides in Japan. However, in a treatment guideline for nonspecialists first published in 2010 by a group of Japanese diabetes specialists [24], biguanides are indicated as first-line therapy, perhaps reflected in our study in the gradual increase in the prevalence of biguanide use in Japan (Fig 1). In addition, for DPP-4 inhibitors, no institutional or economic constraint was present in Japan, which may help to explain the rapid penetration of DPP-4 inhibitors.

In Hong Kong and Taiwan, physicians in general follow the guidelines for treatment of diabetes in the West. In addition, in Hong Kong, newly marketed medications are not included in the drug formulary of public hospitals immediately, and medication cost is not covered by the public health care system at that time. Consequently, we observed low rates of use of DPP-4 inhibitors in early years of approval. Sitagliptin was listed in the public hospital formulary in November 2009 and since then the drug cost has been covered. Therefore, the use of DPP-4 inhibitors increased gradually. In Taiwan, after the approval by Taiwan FDA, a newly launched drug is submitted to the Administration of National Health Insurance to obtain its reimbursement price. The listing of a new drug in the hospital formulary is then acceptable usually in medical centers first followed by other health service providers. Therefore, it takes around 2 to 3 years for a new drug to be accessible nationwide in Taiwan. In addition, physicians in primary care settings had the following constrains, which may have deterred them from prescribing sitagliptin (the only DPP-4 inhibitor available in Taiwan between 2001 and 2010): (1) sitagliptin can be used only when good glucose control has not been achieved by 2 other medication classes including biguanides, sulfonylureas, alpha-glucosidase inhibitors, and meglitinides; (2) it is requested to report the level of HbA1c 3 to 6 months before and after the initiation of sitagliptin treatment and the reason for its use and the disease history of the patient.

The drug price and patients' out-of-pocket expenditure could be also associated with the difference of the penetration pattern of DPP-4 inhibitors between countries. In Hong Kong and Taiwan, all the prescription drugs are covered by the insurance (after the drug is included in the drug formulary) and therefore, the drug price does not directly affect the out-of-pocket expenditure. In Japan, the legacy of physician dispensing may affect the penetration pattern of new drugs. The physician dispensing occurs when prescribing and dispensing are not separated where physicians obtain physician markup that is in general higher for more expensive drugs [25,26]. As the treatment with sitagliptin, the first DPP-4 inhibitor in Japan was more expensive (around $50/month for the typical maintenance dose (100 mg/day)) than the treatment with metformin (around $7/month for the typical maintenance dose (1,500 mg/day)), the physician markup was higher for sitagliptin than metformin. This difference of markup could have worked as one of factors leading to the rapid penetration of DPP-4 inhibitors in Japan. However, it was unlikely that the physician markup had the major impact because the proportion of separation of prescribing and dispensing was attained for more than 60% of drugs dispensed in 2010 when the rapid increase of the prescription of DPP-4 inhibitors began (Fig 2) [27]. In addition, it has been shown that physicians are more responsive to the patient’s out-of-pocket costs (10 to 30% of the health care cost including medication fee) than their own profits from markup [25]. Similarly, in one study in the United States, substantial regional variation in the use of incretin based drugs (mainly sitagliptin introduced in 2006) was observed in Medicare Part D beneficiaries [28]. The regional variation remained even after adjusted for sociodemographics including an indicator of low-income subsidy, indicating that the out-of-pocket expenditure in 2009–2010, which was higher in users of incretin based drugs ($369) than in users of non- incretin based drugs ($151), was probably not an important factor accounting for the regional variation of the penetration pattern of incretin based drugs in the United States.

One strength of the current study is the use of the Common Data Model with a single analytic program developed in the coordinating center. This approach allowed us to retrieve the data in the same manner in different countries while no individual-level data were released from the study sites. Another advantage was the use of large databases, though the sizes differed substantially between countries.

Our study has limitations. First, the databases were not perfectly comparable in terms of contents or patient populations. For example, 3 databases were claims databases and 1 database was an electronic medical record system. However, we carefully mapped local codes for medications and conditions to the standard vocabulary in the Common Data Model to minimize loss of comparability due to different coding systems, and we used age- and sex-standardized prevalence and incidence rates for better comparison. Also, it is likely that the way medications, diagnoses, and other variables were coded differed between countries. To address this problem, we compared patterns of prevalence, incidence, and the proportion of patients with various characteristics between new users of DPP-4 inhibitors and biguanides in each country first and then the pattern of prevalence or incidence in the 7 medication classes or the difference in the proportions between DPP-4 inhibitors and biguanides was compared between the 4 countries.

## Conclusion

We observed a marked difference in the market penetration patterns of a new class of antidiabetic medications, taking DPP-4 inhibitors as an example between 3 Asian countries and the United States. In a country with low penetration (eg, Hong Kong), patients using DPP-4 inhibitors tended to have multiple comorbid conditions, whereas in a country with high penetration (eg, Japan), the proportion of patients with comorbid conditions was similar between new users of DPP-4 inhibitors and new users of biguanides. Differences in treatment guidelines and constraints on the use of the new medication class may partly explain those differences. However, more studies are needed to confirm the reasons for the different penetration patterns and the impact of the different penetration patterns on treatment outcomes of patients with diabetes.

## Supporting information

S1 Table Characteristics of new users of dipeptidyl peptidase-4 inhibitors and biguanides younger than 65 years. (DOCX)

S2 Table Characteristics of new users of dipeptidyl peptidase-4 inhibitors and biguanides 65 years and older. (DOCX)
