# Supplementary material for: Penetration of new antidiabetic medications in East Asian countries and the United States: A cross-national comparative study
Source: PLoS One. 2018 Dec 12;13(12):e0208796. doi: 10.1371/journal.pone.0208796 (PMC6291148; doi:10.1371/journal.pone.0208796)
Supplement: S1 Table — (DOCX) [file pone.0208796.s001.docx]

**S1 Table** **Characteristics of new users of dipeptidyl peptidase-4 inhibitors and biguanides younger than 65 years**

| Characteristic | Taiwan | | | Hong Kong | | | Japan | | | United States | | |
| --- | --- | --- | --- | --- | --- | --- | --- | --- | --- | --- | --- | --- |
|  | DPP-4 Inhibitor (n = 4338) | Biguanide (n = 33,493) | Std Diff | DPP-4 Inhibitor (n = 286) | Biguanide (n = 1561) | Std Diff | DPP-4 Inhibitor (n = 20,652) | Biguanide (n = 12,734) | Std Diff | DPP-4 Inhibitor (n = 7149) | Biguanide (n = 21,050) | Std Diff |
| Men, No. (%) | 2337 (53.9) | 17,771 (53.1) | 0.02 | 157 (54.9) | 834 (53.4) | 0.03 | 15,356 (74.4) | 9210 (72.3) | 0.05 | 3132 (43.8) | 9776 (46.4) | –0.05 |
| Age, mean (SD), y | 53.0 (8.2) | 49.2 (10.7) | 0.35 | 54.4 (8.0) | 53.4 (8.2) | 0.12 | 52.2 (8.4) | 50.1 (9.4) | 0.24 | 53.3 (8.3) | 54.1 (8.0) | –0.10 |
| Pattern, No. (%)^a^ |  |  |  |  |  |  |  |  |  |  |  |  |
| Single | 198 (4.6) | 17,720 (52.9) | –0.97 | 7 (2.4) | 1022 (65.5) | –1.27 | 7622 (36.9) | 3871 (30.4) | 0.14 | 1444 (20.2) | 14,002 (66.5) | –0.93 |
| Dual | 1445 (33.3) | 13,939 (41.6) | –0.17 | 45 (15.7) | 490 (31.4) | –0.35 | 6597 (31.9) | 4773 (37.5) | –0.12 | 3018 (42.2) | 5626 (26.7) | 0.34 |
| Multiple | 2695 (62.1) | 1834 (5.5) | 1.74 | 234 (81.8) | 49 (3.1) | 2.18 | 6433 (31.1) | 4090 (32.1) | –0.02 | 2687 (37.6) | 1422 (6.8) | 0.87 |
| Concurrent antidiabetic medication, No. (%) |  |  |  |  |  |  |  |  |  |  |  |  |
| Alpha-glucosidase inhibitor | 677 (15.6) | 1062 (3.2) | 0.59 | 19 (6.6) | 6 (0.4) | 0.54 | 3736 (18.1) | 2370 (18.6) | –0.01 | 53 (0.7) | 26 (0.1) | 0.11 |
| Biguanide | 2812 (64.8) | — | — | 263 (92.0) | — | — | 6267 (30.3) | — | — | 3636 (50.9) | — | — |
| DPP-4 inhibitor | — | 151 (0.5) | — | — | 21 (1.3) | — | — | 4309 (33.8) | — | — | 4309 (20.5) | — |
| Insulin | 500 (11.5) | 1002 (3.0) | 0.44 | 63 (22.0) | 96 (6.1) | 0.57 | 2042 (9.9) | 1521 (11.9) | –0.06 | 1715 (24.0) | 3087 (14.7) | 0.25 |
| Meglitinide | 316 (7.3) | 1092 (3.3) | 0.21 | 0 | 1 (0.1) | –0.04 | 743 (3.6) | 537 (4.2) | –0.03 | 148 (2.1) | 134 (0.6) | 0.15 |
| Sulfonylurea | 2659 (61.3) | 13,449 (40.2) | 0.43 | 214 (47.8) | 459 (29.4) | 0.94 | 6052 (29.3) | 3742 (29.4) | 0.00 | 1954 (27.3) | 3018 (14.3) | 0.34 |
| Thiazolidinedione | 826 (19.0) | 1039 (3.1) | 0.73 | 16 (5.6) | 6 (0.4) | 0.48 | 3207 (15.5) | 1790 (14.1) | 0.04 | 1593 (22.3) | 1775 (8.4) | 0.43 |
| Comorbid conditions, No. (%) |  |  |  |  |  |  |  |  |  |  |  |  |
| Asthma | 164 (3.8) | 1289 (3.8) | 0.00 | 1 (0.3) | 1 (0.1) | 0.06 | 1872 (9.1) | 1125 (8.8) | 0.01 | 1293 (18.1) | 3691 (17.5) | 0.02 |
| Atrial fibrillation | 36 (0.8) | 168 (0.5) | 0.04 | 1 (0.3) | 5 (0.3) | 0.00 | 344 (1.7) | 143 (1.1) | 0.05 | 350 (4.9) | 863 (4.1) | 0.04 |
| COPD | 62 (1.4) | 561 (1.7) | –0.02 | 0 | 1 (0.1) | –0.04 | 1959 (9.5) | 1124 (8.8) | 0.02 | 539 (7.5) | 1573 (7.5) | 0.00 |
| Dementia | 12 (0.3) | 114 (0.3) | 0.00 | 0 | 3 (0.2) | –0.05 | 19 (0.1) | 7 (0.1) | 0.00 | 70 (1.0) | 234 (1.1) | –0.01 |
| Depression | 176 (4.1) | 1212 (3.6) | 0.03 | 0 | 13 (0.8) | –0.10 | 1069 (5.2) | 639 (5.0) | 0.01 | 1021 (14.3) | 3184 (15.1) | –0.02 |
| Epilepsy | 25 (0.6) | 176 (0.5) | 0.01 | 0 | 0 | — | 313 (1.5) | 188 (1.5) | 0.00 | 262 (3.7) | 902 (4.3) | –0.03 |
| Heart failure | 167 (3.8) | 617 (1.8) | 0.14 | 3 (1.0) | 0 | 0.25 | 1940 (9.4) | 1071 (8.4) | 0.03 | 1145 (16.0) | 2384 (11.3) | 0.14 |
| Hyperlipidemia | 2569 (59.2) | 12,345 (36.9) | 0.46 | 8 (2.8) | 24 (1.5) | 0.10 | 12,915 (62.5) | 7435 (58.4) | 0.08 | 5740 (80.3) | 13,964 (66.3) | 0.31 |
| Hypertension | 2221 (51.2) | 12,047 (36.0) | 0.31 | 9 (3.1) | 70 (4.5) | –0.07 | 10,392 (50.3) | 5658 (44.4) | 0.12 | 6124 (85.7) | 15,631 (74.3) | 0.27 |
| Malignant neoplasm | 167 (3.8) | 987 (2.9) | 0.05 | 2 (0.7) | 14 (0.9) | –0.02 | 3990 (19.3) | 2283 (17.9) | 0.04 | 470 (6.6) | 1253 (6.0) | 0.03 |
| Mood disorder | 188 (4.3) | 1351 (4.0) | 0.02 | 0 | 13 (0.8) | –0.10 | 1139 (5.5) | 685 (5.4) | 0.00 | 1383 (19.3) | 4516 (21.5) | –0.05 |
| Myocardial infarction | 62 (1.4) | 298 (0.9) | 0.05 | 1 (0.3) | 4 (0.3) | 0.0 | 841 (4.1) | 462 (3.6) | 0.03 | 144 (2.0) | 303 (1.4) | 0.05 |
| Parkinson disease | 10 (0.2) | 65 (0.2) | 0.00 | 0 | 2 (0.1) | –0.03 | 82 (0.4) | 43 (0.3) | 0.02 | 32 (0.4) | 123 (0.6) | –0.03 |
| Pneumonia | 150 (3.5) | 881 (2.6) | 0.06 | 1 (0.3) | 6 (0.4) | –0.02 | 804 (3.9) | 462 (3.6) | 0.02 | 116 (1.6) | 303 (1.7) | –0.01 |
| Renal failure | 242 (5.6) | 366 (1.1) | 0.36 | 2 (0.7) | 2 (0.1) | 0.13 | 273 (1.3) | 106 (0.8) | 0.05 | 603 (8.4) | 803 (3.8) | 0.21 |
| Rheumatoid arthritis | 40 (0.9) | 235 (0.7) | 0.02 | 0 | 0 | — | 591 (3.8) | 289 (3.6) | 0.04 | 270 (3.8) | 764 (3.6) | 0.01 |
| Schizophrenia | 47 (1.1) | 401 (1.2) | –0.01 | 0 | 2 (0.1) | –0.03 | 269 (1.3) | 178 (0.8) | –0.01 | 491 (6.9) | 1809 (8.6) | –0.06 |
| Concurrent medications, No. (%) |  |  |  |  |  |  |  |  |  |  |  |  |
| Antiarrhythmic | 86 (2.0) | 534 (1.6) | 0.03 | 1 (0.3) | 6 (0.4) | 0.02 | 2622 (12.7) | 1402 (11.0) | 0.05 | 385 (5.4) | 790 (3.8) | 0.08 |
| Antidementia | 71 (1.6) | 343 (1.0) | 0.06 | 0 | 1 (0.1) | 0.02 | 7 (< 0.1) | 3 (< 0.1) | 0.00 | 88 (1.2) | 198 (0.9) | 0.03 |
| Antidepressant | 360 (8.3) | 2166 (6.5) | 0.07 | 8 (2.8) | 60 (3.8) | 0.02 | 730 (3.5) | 486 (3.8) | –0.02 | 3221 (45.1) | 9227 (43.8) | 0.03 |
| Anti-Parkinson | 112 (2.6) | 1037 (3.1) | –0.03 | 0 | 28 (1.8) | –0.15 | 121 (0.6) | 69 (0.5) | 0.01 | 527 (7.4) | 1624 (7.7) | –0.01 |
| Antipsychotic | 268 (6.2) | 2395 (7.2) | –0.04 | 13 (4.5) | 86 (5.5) | –0.04 | 599 (2.9) | 369 (2.9) | 0.00 | 1505 (21.1) | 5108 (24.3) | –0.08 |
| Benzodiazepine | 665 (15.3) | 4483 (13.4) | 0.06 | 15 (5.2) | 70 (4.5) | 0.03 | 2106 (10.2) | 1098 (8.6) | 0.05 | 919 (12.9) | 2229 (10.6) | 0.07 |
| β-Blocker | 1256 (29.0) | 7030 (21.0) | 0.19 | 81 (28.3) | 285 (18.3) | 0.25 | 1730 (8.4) | 837 (6.6) | 0.07 | 2697 (37.7) | 6137 (29.2) | 0.18 |
| Calcium channel blocker | 1560 (36.0) | 7657 (22.9) | 0.31 | 89 (31.1) | 368 (23.6) | 0.17 | 4933 (23.9) | 2459 (19.3) | 0.11 | 1659 (23.2) | 3662 (17.4) | 0.15 |
| COPD medication | 1373 (31.7) | 10,844 (32.4) | –0.01 | 8 (2.8) | 51 (3.3) | –0.03 | 4561 (22.1) | 2639 (20.7) | 0.03 | 2601 (36.4) | 6777 (32.2) | 0.09 |
| Diuretic | 1106 (25.5) | 4364 (13.0) | 0.36 | 27 (9.4) | 56 (3.6) | 0.28 | 1085 (5.3) | 505 (4.0) | 0.06 | 3520 (49.2) | 8320 (39.5) | 0.20 |
| Non-statin lipid-lowering drug | 649 (15.0) | 1979 (5.9) | 0.36 | 21 (7.3) | 28 (1.8) | 0.34 | 1334 (6.5) | 697 (5.5) | 0.04 | 1028 (14.4) | 1671 (7.9) | 0.22 |
| NSAID | 2444 (56.3) | 19477 (58.2) | –0.04 | 20 (7.0) | 162 (10.4) | –0.11 | 5766 (27.9) | 3328 (26.1) | 0.04 | 2039 (28.5) | 5617 (26.7) | 0.04 |
| RAS inhibitor | 624 (14.4) | 3364 (10.0) | 0.14 | 127 (44.4) | 198 (12.7) | 0.83 | 790 (3.8) | 414 (3.3) | 0.03 | 3398 (47.5) | 7034 (33.4) | 0.29 |
| Statin | 1834 (42.3) | 3710 (11.1) | 0.88 | 112 (39.2) | 225 (14.4) | 0.64 | 6423 (31.1) | 3265 (25.6) | 0.12 | 4197 (58.7) | 8275 (39.3) | 0.39 |
| Vitamin K antagonist | 47 (1.1) | 123 (0.4) | 0.10 | 5 (1.7) | 8 (0.5) | 0.14 | 272 (1.3) | 99 (0.8) | 0.05 | 371 (5.2) | 934 (4.4) | 0.04 |

Abbreviations: Std Diff, standardized difference; COPD, chronic obstructive pulmonary disease; DPP-4, dipeptidyl peptidase-4; NSAID, nonsteroidal anti-inflammatory drug; RAS, renin-angiotensin system.

^a^ Single use refers to new use of a DPP-4 inhibitor or biguanide without concurrent use or initiation of another antidiabetic medication. Dual use refers to new use of a DPP-4 inhibitor or biguanide with concurrent use or initiation of 1 other antidiabetic medication. Multiple use refers to new use of a DPP-4 inhibitor or biguanide with concurrent use or initiation of 2 or more other antidiabetic medications.
