# Supplementary material for: Penetration of new antidiabetic medications in East Asian countries and the United States: A cross-national comparative study
Source: PLoS One. 2018 Dec 12;13(12):e0208796. doi: 10.1371/journal.pone.0208796 (PMC6291148; doi:10.1371/journal.pone.0208796)
Supplement: S2 Table — (DOCX) [file pone.0208796.s002.docx]

**S2 Table** **Characteristics of new users of dipeptidyl peptidase-4 inhibitors and biguanides younger than 65 years**

| Characteristic | Taiwan | | | Hong Kong | | | Japan | | | United States | | |
| --- | --- | --- | --- | --- | --- | --- | --- | --- | --- | --- | --- | --- |
|  | DPP-4 Inhibitor (n = 3159) | Biguanide (n = 14,954) | Std Diff | DPP-4 Inhibitor (n = 244) | Biguanide (n = 1106) | Std Diff | DPP-4 Inhibitor (n = 2945) | Biguanide (n = 1270) | Std Diff | DPP-4 Inhibitor (n = 26,271) | Biguanide (n = 55,082) | Std Diff |
| Men, No. (%) | 1373 (43.5) | 6844 (45.8) | –0.05 | 92 (37.7) | 527 (47.6) | –0.20 | 1631 (55.4) | 692 (54.5) | 0.02 | 9490 (36.1) | 20,444 (37.1) | –0.02 |
| Age, mean (SD), y | 73.7 (6.4) | 73.1 (6.1) | 0.10 | 74.8 (6.7) | 74.7 (7.1) | 0.01 | 68.9 (3.2) | 68.6 (3.2) | 0.24 | 75.7 (7.1) | 74.9 (7.1) | 0.11 |
| Pattern, No. (%)^a^ |  |  |  |  |  |  |  |  |  |  |  |  |
| Single | 233 (7.4) | 7781 (52.0) | –0.90 | 10 (4.1) | 654 (59.1) | –1.10 | 1041 (35.3) | 247 (19.4) | 0.35 | 6218 (23.7) | 34,419 (62.5) | –0.78 |
| Dual | 1063 (33.6) | 6303 (42.1) | –0.17 | 46 (18.9) | 416 (37.6) | –0.39 | 997 (33.9) | 494 (38.9) | –0.10 | 11,371 (43.3) | 16,707 (30.3) | 0.27 |
| Multiple | 1863 (59.0) | 870 (5.8) | 1.49 | 190 (77.9) | 51 (4.6) | 1.91 | 907 (30.8) | 529 (41.7) | –0.23 | 8682 (33.0) | 3956 (7.2) | 0.71 |
| Concurrent antidiabetic medication, No. (%) |  |  |  |  |  |  |  |  |  |  |  |  |
| Alpha-glucosidase inhibitor | 608 (19.2) | 554 (3.7) | 0.63 | 15 (6.1) | 9 (0.8) | 0.40 | 618 (21.0) | 321 (25.3) | –0.10 | 243 (0.9) | 143 (0.3) | 0.09 |
| Biguanide | 1688 (53.4) | — | — | 192 (78.7) | — | — | 682 (23.2) | — | — | 11277 (42.9) | — | — |
| DPP-4 inhibitor | — | 69 (0.5) | — | — | 12 (1.1) | — | — | 465 (36.6) | — | — | 1931 (3.5) | — |
| Insulin | 414 (13.1) | 518 (3.5) | 0.43 | 46 (18.9) | 76 (6.9) | 0.42 | 318 (10.8) | 179 (14.1) | –0.10 | 3990 (15.2) | 5602 (10.2) | 0.16 |
| Meglitinide | 354 (11.2) | 613 (4.1) | 0.32 | 1 (0.4) | 2 (0.2) | 0.04 | 114 (3.9) | 54 (4.3) | –0.02 | 989 (3.8) | 621 (1.1) | 0.19 |
| Sulfonylurea | 1860 (58.9) | 5974 (39.9) | 0.38 | 192 (78.7) | 418 (37.8) | 0.82 | 968 (32.9) | 493 (38.8) | –0.12 | 8724 (33.2) | 11,405 (20.7) | 0.29 |
| Thiazolidinedione | 493 (15.6) | 401 (2.7) | 0.60 | 13 (5.3) | 6 (0.5) | 0.41 | 439 (14.9) | 213 (16.8) | –0.05 | 5304 (20.2) | 5332 (9.7) | 0.31 |
| Comorbid conditions, No. (%) |  |  |  |  |  |  |  |  |  |  |  |  |
| Asthma | 200 (6.3) | 1227 (8.2) | –0.07 | 0 | 12 (1.1) | –0.12 | 285 (9.7) | 123 (9.7) | 0.00 | 2321 (8.8) | 4982 (9.0) | –0.01 |
| Atrial fibrillation | 126 (4.0) | 473 (3.2) | 0.04 | 9 (3.7) | 19 (1.7) | 0.14 | 147 (5.0) | 54 (4.3) | 0.03 | 4027 (15.3) | 7038 (12.8) | 0.07 |
| COPD | 168 (5.3) | 1270 (8.5) | –0.12 | 1 (0.4) | 19 (1.7) | –0.11 | 405 (13.8) | 142 (11.2) | 0.08 | 1722 (6.6) | 3735 (6.8) | –0.01 |
| Dementia | 166 (5.3) | 596 (4.0) | 0.06 | 0 | 9 (0.8) | –0.10 | 55 (1.9) | 14 (1.1) | 0.06 | 1570 (6.0) | 3292 (6.0) | 0.00 |
| Depression | 160 (5.1) | 648 (4.3) | 0.04 | 2 (0.8) | 5 (0.5) | 0.04 | 135 (4.6) | 53 (4.2) | 0.02 | 1302 (5.0) | 2610 (4.7) | 0.01 |
| Epilepsy | 16 (0.5) | 115 (0.8) | –0.04 | 0 | 2 (0.2) | –0.05 | 53 (1.8) | 24 (1.9) | –0.01 | 291 (1.1) | 616 (1.1) | 0.00 |
| Heart failure | 277 (8.8) | 1078 (7.2) | 0.06 | 16 (6.6) | 26 (2.4) | 0.24 | 543 (18.4) | 181 (14.3) | 0.11 | 6022 (22.9) | 8630 (15.7) | 0.19 |
| Hyperlipidemia | 1524 (48.2) | 5139 (34.4) | 0.29 | 21 (8.6) | 43 (3.9) | 0.22 | 1968 (66.8) | 837 (65.9) | 0.02 | 22,463 (85.5) | 42,907 (77.9) | 0.19 |
| Hypertension | 2147 (68.0) | 8830 (59.0) | 0.18 | 49 (20.1) | 118 (10.7) | 0.29 | 1998 (67.8) | 826 (65.0) | 0.06 | 23,988 (91.3) | 47,541 (86.3) | 0.15 |
| Malignant neoplasm | 269 (8.5) | 1011 (6.8) | 0.07 | 5 (2.0) | 22 (2.0) | 0.00 | 955 (32.4) | 397 (31.3) | 0.02 | 3880 (14.8) | 72,70 (13.2) | 0.05 |
| Mood disorder | 171 (5.4) | 696 (4.7) | 0.03 | 2 (0.8) | 5 (0.5) | 0.04 | 142 (4.8) | 55 (4.3) | 0.02 | 1434 (5.5) | 2907 (5.3) | 0.01 |
| Myocardial infarction | 97 (5.4) | 31 (4.7) | 0.08 | 3 (1.2) | 10 (0.9) | 0.03 | 199 (6.8) | 74 (5.8) | 0.04 | 669 (2.5) | 1106 (2.0) | 0.03 |
| Parkinson disease | 56 (1.8) | 260 (1.7) | 0.01 | 0 | 3 (0.3) | –0.06 | 27 (0.9) | 5 (0.4) | 0.06 | 438 (1.7) | 815 (1.5) | 0.02 |
| Pneumonia | 206 (6.5) | 986 (6.6) | 0.00 | 8 (3.3) | 31 (2.8) | 0.03 | 173 (5.9) | 59 (4.6) | 0.06 | 554 (2.1) | 1062 (1.9) | 0.01 |
| Renal failure | 349 (11.0) | 474 (3.2) | 0.37 | 11 (4.5) | 9 (0.8) | 0.31 | 79 (2.7) | 14 (1.1) | 0.11 | 2625 (10.0) | 2335 (4.2) | 0.24 |
| Rheumatoid arthritis | 42 (1.3) | 189 (1.3) | 0.00 | 0 | 0 | — | 122 (4.1) | 53 (4.2) | –0.01 | 929 (3.5) | 1817 (3.3) | 0.01 |
| Schizophrenia | 7 (0.2) | 38 (0.3) | –0.02 | 0 | 4 (0.4) | –0.07 | 29 (1.0) | 9 (0.7) | 0.03 | 160 (0.6) | 455 (0.8) | –0.02 |
| Concurrent medication, No. (%) |  |  |  |  |  |  |  |  |  |  |  |  |
| Antiarrhythmic | 136 (4.3) | 537 (3.6) | 0.04 | 6 (2.5) | 3 (0.3) | 0.27 | 614 (20.8) | 240 (18.9) | 0.05 | 1718 (6.5) | 2249 (4.1) | 0.11 |
| Antidementia | 120 (3.8) | 518 (3.5) | 0.02 | 4 (1.6) | 11 (1.0) | 0.06 | 26 (0.9) | 4 (0.3) | 0.07 | 1702 (6.5) | 3087 (5.6) | 0.04 |
| Antidepressant | 416 (13.2) | 1501 (10.0) | 0.10 | 15 (6.1) | 47 (4.2) | 0.09 | 81 (2.8) | 31 (2.4) | 0.02 | 6541 (24.9) | 12,942 (23.5) | 0.03 |
| Anti-Parkinson | 142 (4.5) | 733 (4.9) | –0.02 | 1 (0.4) | 16 (1.4) | –0.09 | 33 (1.1) | 6 (0.5) | 0.06 | 1019 (3.9) | 1852 (3.4) | 0.03 |
| Antipsychotic | 285 (9.0) | 1252 (8.4) | 0.02 | 19 (7.8) | 92 (8.3) | –0.02 | 83 (2.8) | 31 (2.4) | 0.02 | 1505 (5.7) | 3066 (5.6) | 0.00 |
| Benzodiazepine | 636 (20.1) | 2725 (18.2) | 0.05 | 9 (3.7) | 37 (3.3) | 0.02 | 477 (16.2) | 178 (14.0) | 0.06 | 2119 (8.1) | 3452 (6.3) | 0.07 |
| β-Blocker | 1248 (39.5) | 4824 (32.3) | 0.15 | 111 (45.5) | 328 (29.7) | 0.34 | 397 (13.5) | 145 (11.4) | 0.06 | 12,758 (48.6) | 22407 (40.7) | 0.16 |
| Calcium channel blocker | 1718 (54.4) | 6817 (45.6) | 0.18 | 125 (51.2) | 490 (44.3) | 0.14 | 1148 (39.0) | 372 (29.3) | 0.20 | 8536 (32.5) | 14,763 (26.8) | 0.13 |
| COPD medication | 1138 (36.0) | 6106 (40.8) | –0.10 | 16 (6.6) | 72 (6.5) | 0.00 | 818 (27.8) | 297 (23.4) | 0.10 | 6707 (25.5) | 13,137 (23.8) | 0.04 |
| Diuretic | 1431 (45.3) | 4577 (30.6) | 0.31 | 58 (23.8) | 136 (12.3) | 0.33 | 285 (9.7) | 89 (7.0) | 0.09 | 14,780 (56.3) | 26,785 (48.6) | 0.15 |
| Non-statin lipid-lowering drug | 278 (8.8) | 787 (5.3) | 0.15 | 8 (3.3) | 18 (1.6) | 0.12 | 123 (4.2) | 38 (3.0) | 0.06 | 2278 (8.7) | 3295 (6.0) | 0.11 |
| NSAID | 1864 (59.0) | 9429 (63.1 | –0.08 | 14 (5.7) | 104 (9.4) | –0.13 | 1074 (36.5) | 393 (30.9) | 0.12 | 5030 (19.1) | 9745 (17.7) | 0.04 |
| RAS inhibitor | 493 (15.6) | 2597 (17.4) | –0.05 | 118 (48.4) | 277 (25.0) | 0.51 | 171 (5.8) | 65 (5.1) | 0.03 | 11,713 (44.6) | 20,799 (37.8) | 0.14 |
| Statin | 1381 (43.7) | 2395 (16.0) | 0.68 | 123 (50.4) | 308 (27.8) | 0.48 | 1158 (39.3) | 450 (35.4) | 0.08 | 16,984 (64.6) | 28,113 (51.0) | 0.27 |
| Vitamin K antagonist | 64 (2.0) | 187 (1.3) | 0.06 | 6 (2.5) | 23 (2.1) | 0.03 | 105 (3.6) | 41 (3.2) | 0.02 | 2658 (10.1) | 4762 (8.6) | 0.05 |

Abbreviations: Std Diff, standardized difference; COPD, chronic obstructive pulmonary disease; DPP-4, dipeptidyl peptidase-4; NSAID, nonsteroidal anti-inflammatory drug; RAS, renin-angiotensin system.

^a^ Single use refers to new use of a DPP-4 inhibitor or biguanide without concurrent use or initiation of another antidiabetic medication. Dual use refers to new use of a DPP-4 inhibitor or biguanide with concurrent use or initiation of 1 other antidiabetic medication. Multiple use refers to new use of a DPP-4 inhibitor or biguanide with concurrent use or initiation of 2 or more other antidiabetic medications.
